# Maximizing the cost benefit of physics residency interview

**DOI:** 10.1002/acm2.12031

**Published:** 2017-02-03

**Authors:** Justus Adamson, Sonja Dieterich, Yi Rong

**Affiliations:** ^1^ Department of Radiation Oncology Duke University Medical Center Durham NC 27712 USA; ^2^ Department of Radiation Oncology University of California Davis Comprehensive Cancer Center Sacramento CA 95718 USA

## 1 Introduction

Dr. Justus Adamson has brought up a discussion on AAPM website regarding how to maximize the cost‐benefit for our current physics residency interview process. It has been an ongoing discussion and had drawn continuous attentions. The main question is that whether the quality of the interview process will be compromised if we seek out other alternatives other than the traditional face‐to‐face meeting. Herein, we invited Dr. Justus Adamson, a resident graduate from Duke University, to debate with Dr. Sonja Dieterich, the resident program director from University of California Davis (UCD) on this topic.

Dr. Justus Adamson is an Assistant Professor in the Department of Radiation Oncology at Duke University. He completed a PhD in Medical Physics from Wayne State University in 2009, and residency training at Duke University in 2011. He is board certified by the American Board of Radiology in Therapeutic Radiological Physics and currently serves on the AAPM International Information Subcommittee. He has active research efforts in clinical radiation oncology physics, with over 30 peer reviewed publications.

Dr. Sonja Dieterich is a Professor in the Department of Radiation Oncology at UCD. She completed her PhD in Nuclear Physics from Rutgers University in 2002, and postdoctoral training at Georgetown University in 2003. Dr. Dieterich is board certified by the American Board of Radiology (ABR) in Therapeutic Radiological Physics and currently serves as the physics residency co‐chair at UCD, as well as the chair of the ABR Initial Certification Part II Therapy Exam Committee. In addition to her clinical work, Dr. Dieterich performs joint research work with the UCD Veterinary Radiation Oncology group.

## 2 Opening statement

### 2.A Dr. Justus Adamson

Prior to the implementation of standardized residencies, the application process for medical physics trainees was similar to that of a traditional job interview. In contrast, the interview process now mirrors that of physician residencies, with programs inviting applicants for onsite interviews each spring. As it is in each program's interest to ensure a match with the best qualified candidate(s), there are often many more applicants interviewed than positions to be filled. Similarly, applicants have equal (if not greater) incentive to ensure a match, thus they interview at numerous institutions. Because of the number of applicants being interviewed, the result is that the expense is transferred to the applicants; hence many incur hefty traveling expenses especially for a student budget. The question I raise is this: is this application process really the optimal and most efficient solution for both the residency programs and applicants?

The status quo is easily justified; after all, it is modeled after the physician residency application and match process with its 60+ year history.^1^ The coordinated match program itself is an improvement upon the prior uncoordinated system.^2^ Disadvantages of the current system such as financial costs to applicants are offset by the promise of a formal accredited training program and subsequent entrance into a rewarding profession. Some programs even offer limited support to applicants such as hotel expenses or a travel voucher. Further support beyond this is hard to justify, given that it would constitute a more generous offering for physics resident applicants than their physician counterparts receive.

Yet still, is this the most efficient solution? These justifications do not preclude the possibility of a better, more efficient process. The residency match paradigm predates the digital age we live in. Nowadays the technological capability to connect to others remotely is boundless, yet applicants still physically trek across the country for face to face interviews. In addition to the other CAMPEP requirements, must we also require that applicants physically demonstrate on a national scale the optimized solution to the traveling salesman problem?^3^

Some excellent gains are already being made by the AAPM such as the recently implemented residency fair at the annual meeting. Yet many possibilities still exist that could be explored to make this application process more efficient. One possibility is to replace on‐site interviews by expanding the residency fair to also include face to face interviews in one centralized meeting location. This could be supplemented with better utilization of web conferencing technology for group interviews with staff who are not in attendance. Arguments against such an approach are the inopportune timing (interviews in spring vs. annual meeting in summer) and that the applicants would lose the opportunity to see the training programs’ environment and facilities. However, these are mostly solvable problems. For instance, using the Spring Clinical Meeting for centralized interviews might better align with the current interview process, and there are now plenty of technologies available to virtually showcase facilities. Nuances about the department such as how well integrated the physicists are with the rest of the clinic, whether the work environment is a positive one, etc., could be gauged by applicants through conversations with current residents. Furthermore, a central interview location would provide a better environment to showcase applicants’ research (for instance via a poster session). These suggestions are only a few of many possibilities. I do not pretend to know what the optimal process is that would meet the need of all residency programs and applicants; however, I do firmly believe that improvements could be made with our collective talent focused on this topic. It's important to note that significant improvements to the status quo won't happen on their own. Residency programs have little impetus to change the current system since it inconveniences the applicant rather than the programs. Thus only a collective effort by the associated professional organizations will bring it about.

We as physicists are ideally situated to find a better solution. Consider the fact that the physician interview and match process is generic to all medical specialties, thus large‐scale changes to it are difficult or impossible to enact, given the numerous specialties and corresponding professional organizations that are involved. In contrast, we are a relatively small organization with relatively few accredited programs; this represents the ideal climate to test and implement better and innovative solutions. Indeed, as physicists, innovation is a key principle that we bring to the table.

### 2.B Dr. Sonja Dieterich

Asking graduate students to attend in‐person residency interviews during their peak thesis writing time is a significant burden both in time and money. On the interviewer's side, spending three full workdays on the interview process is a significant time burden on clinical physicists, and cost to the department. Therefore, our residency committee put a lot of thought into how to make the interview process both effective and efficient. We conducted phone interviews in the initial years of our program, but we found them to be insufficient to evaluate the candidates. We also believe there could be bias introduced by phone interviews, for example, because of language issues on both ends of the line or increased nerves when the candidate is unable to see the interviewer's response to them. After several instances of a candidate making a very different impression in person compared to the phone interview (in either direction), we decided to change to a face‐to‐face interview process.

By visiting the program in person, the candidates will get a much better picture of who we are as a department, both for our equipment as well as the personalities of the faculty involved in teaching. Some candidates have been positively surprised; others decided that we might not be the best fit for them. As an English‐as‐second‐language (ESL) speaker myself, I found the additional information of body language, etc. during a face‐to‐face interview to be very helpful to bridge potential language barriers. A longer interview process, even though it is tiring by the end of the day, also helps more introverted candidates. Over the time of an interview day, more introverted candidates often start to relax and are able to engage better with the interviewers compared to the start of the day. Each interview cycle, we consistently see several candidates who need one or two “warm‐up” interviews before they were able to shine.

It is also very important to us that the current residents meet the candidates in person. As a smaller program with two physics residents and six medical residents sharing one resident's room, residents will spend a lot of time working with each other. While it is an important professional skill to work with diverse colleagues, we want to be mindful of how good working relationships among residents go a long way to build a good support network between them.

Another question to consider is if it is really necessary for programs to invite a large number of candidates rather than just inviting the top five candidates. As a program director, I have to weigh the opportunity cost of faculty spending time interviewing many candidates vs. the risk of not matching. After having the experience of scrambling one year after our match had to retract the candidacy for personal reasons, our program does err on the side of caution for the number of candidates we invite.

Still, the cumulative cost of interviewing for residents is of great concern to me, because I worry that we are creating barriers for bright students with limited economic resources to gain access to residencies. Pricing good candidates out of the interview process would be a loss to our field which we cannot afford. Our physics residency committee has discussed a scenario in which a highly qualified candidate expresses interest in an interview, but shares with us they would not be able to attend in person because of financial constraints. While we might be able to set up a video conference call (our IT department does not allow the use of Skype or other services through the hospital network), how would we make sure that the difference in interview process between candidates does not create unfair bias, be it explicit or implicit?

Unfortunately the fiscal constraints we face as a public university hospital do not allow us to offer financial assistance for attending interviews other than providing meals during the day and accessing to discounted hotel rates. In recent years, we have also worked to coordinate interview dates with other residency programs in our state to allow candidates to minimize travel time and expenses.

In summary, I believe the benefits of the in‐person interview process currently outweigh the costs. As the supply of residency positions and the size of the candidate pool changes, the cost‐benefit analysis might well change. Changes in technology, especially improved quality for videoconferencing, might be another factor driving future changes.

## 3 Rebuttal

### 3.A Dr. Justus Adamson

As pointed out by Dr. Dieterich, a key question is whether the benefits of on‐site interviews outweigh the costs. I agree that there are clear advantages to on‐site interviews, and likely many physicists will be skeptical of proposals to eliminate it. Either way, whether or not on‐site interviews are worth the travel expense is really a question for the applicants, who are the ones currently shouldering the bulk of the cost.

However, we could still improve upon the current process even if collectively we are not ready to move away from on‐site interviews. The most straightforward improvement would be better coordination of interview schedules, such as coordinating interview dates by geographic location and eliminating conflicting interview dates. Some coordination already occurs on a limited scale and is common practice for many physician training programs. Another potential improvement would be minimizing travel expenses by inviting fewer applicants for on‐site interviews. As mentioned above, the impediment to de‐escalating the number of applicants interviewed per residency slot is the institutions’ understandable incentive to secure a match with highly qualified candidate(s). Given this fact, I imagine that successful de‐escalation would likely require something along the lines of a coordinated interim match, possibly based on remote (video conference) or centralized interviews (in conjunction with a major meeting). Remote or centralized interviews as a middle step between initially evaluating applications and inviting candidates to onsite interviews could benefit both parties as they give programs a chance to screen out applications that may look great on paper, but ultimately aren't a good fit. Likewise, candidates could get an initial taste of the program by talking to faculty which helps in prioritizing visits. The end result would be a higher percentage of onsite candidates being acceptable and the candidates choosing to interview with programs that are a better fit.

But would the advantages of on‐site interviews really be lost under a new program? I have participated in both phone and video conference interviews, and there is a big difference between them. A high‐quality video conference interview between training faculty on one end and an applicant on the other can arguably be as useful as if the interview had taken place face to face. Furthermore, face‐to‐face interviews need not necessarily be eliminated if a centralized interview location is utilized. It is important to note that there is precedent for such an arrangement; for example, the Loyola Patent Law Interview Program^4^ is a two‐day event held in Chicago each year that brings together patent law employers and law students from across the country to interview for summer associate positions and post‐graduate employment. Students register, submit resumes and transcripts, and bid on interviews with nation‐wide employers. Participating employers review the materials submitted and choose students they are interested in interviewing. What is more difficult to replace is the ability of applicants to assess the work culture; but I imagine that little would be lost if one‐on‐one conversations with current physics residents remains a component of the interview process.

### 3.B Dr. Sonja Dieterich

It is challenging to write a rebuttal when I find my opponent's opening statement to be less parallel opposed but more like a wedged pair. I agree with Dr. Adamson that the current process is better than the previous system, aligned with what physicians have been practicing for years, and yet not ideal because of the fiscal and time burden on candidates. The idea of a centralized interview process at the AAPM Spring Clinical meeting has come to my mind as well; there is precedence in the interview sessions for graduating physician residents at ASTRO. If the Spring Clinical meeting were consistently in February, there would be enough time to execute the match and process all administrative paperwork (including visas for our international residents) in time for a July 1 start. There are two drawbacks: (a) we would need to find a volunteer team and funding for administrative support to make it happen, and (b) likely only the residency director and vice‐director could interview, because meeting attendance per physics group is limited. With one or two years lead, the first problem could be overcome by raising the MP‐RAP application fee, which would still be lower cost to applicants than the current travel expense. The second drawback, limited number of interviewers, requires more thought to solve. For the departments I have worked in, we have traditionally placed emphasis on all physics faculty to have equitable input in the residency selection to get their buy‐in for maximum support of our residents. Web conferencing seems a relatively easy solution, but in reality the quality of video calls is still all over the map. Even when using well established commercial web conferencing services from office phones, call quality can range from excellent to almost impossible to communicate. I still think there is a considerable amount of important information about a department that the candidate would not be able to get from a video call, but this might be an acceptable compromise given the cost of travel.

Clearly, the optimum solution to how we conduct the interview process will be a function of time. We have reached a temporary equilibrium, with a clear need for further adjustment in the future. This is how I envision us moving forward: At this time, we need to give the match system a few more years to stabilize. Not all programs participate in the match yet, and employers have not yet adjusted recruitment to the residency graduation dates. While we all adjust to the match process using the current on‐site interview process, smaller subgroups of residency programs could start working on single‐site interview locations either coupled to the Spring Clinical Meeting or local chapter meetings. On the basis of the lessons learned from these smaller joint interview groups, we can decide if centralized interview is indeed an effective alternative and develop a plan for implementation. In parallel, web conferencing technologies should improve in call quality consistency, making it a more attractive alternative to face‐to‐face interviews. A panel session on interview process design should become part of the annual SDAMPP meeting going forward, including resident representatives.

## 4 Conflict of interest

All authors have no conflict of interests to disclose.
